# Anti-Metastatic Potential of a Novel Xanthone Sourced by *Swertia chirata* Against *In Vivo* and *In Vitro* Breast Adenocarcinoma Frameworks

**DOI:** 10.31557/APJCP.2020.21.10.2865

**Published:** 2020-10

**Authors:** Atish Barua, Pritha Choudhury, Suvra Mandal, Chinmay Kumar Panda, Prosenjit Saha

**Affiliations:** 1 *Department of Cancer Chemoprevention, Chittaranjan National Cancer Institute, West Bengal, India. *; 2 *National Research Institute of Ayurvedic Drug Development, 4 Minerva Road, CN Block, Sector V, Bidhannagar, Kolkata, West Bengal, India. *; 3 *Department of Oncogenne regulation, Chittaranjan National Cancer Institute, West Bengal, India. *

**Keywords:** Breast cancer, metastasis, xanthone

## Abstract

**Background:**

The Anticancer property of *Swertia chirata* has been well established. It forms a rich source of compounds to which its anticancer property can be attributed, among the compounds found in S. chirata xanthones form an important group. Among the most abundant xanthones found in S. chirata, 1,5,8-trihydroxy-3-methoxy xanthone (TMX) was found to be most effective. As metastasis is the underlying cause of most cancer-related deaths, in this study, we evaluated the anti-metastatic potential of TMX against adenocarcinoma both *in vivo* and *in vitro*.

**Materials and Methods:**

*In vivo* anti-metastatic potential was proved by histological evidence of different organs, giemsa staining of bone marrow, subcutaneous re-injection of the aberrant bone marrow cells into the right flank of the mice to observe the formation of tumors and analyzing the markers related to metastasis by immunohistochemistry (IHC) and western blot. *In vitro* validation of anti-metastatic potential was carried out against human breast adenocarcinoma cell line MCF-7 by primarily analyzing the migratory property of cells through scratch wound healing assay and the ability of cells to form colonies. The re-validation part was performed by western blot of markers related to metastasis and real-time analysis of EMT related markers.

**Results:**

*In vivo*, TMX treatment restricted metastasis of EAC induced solid tumor to liver, lung, bone marrow, and validation of this finding was achieved by down regulation of metastatic and EMT markers. *In vitro*, TMX treatment restricted migratory and colony forming ability of MCF-7 cells by down regulating metastatic and EMT markers.

**Conclusion:**

It was proved from our study that TMX treatment successfully reduced the metastatic potential of EAC induced solid tumor, with *in vitro* validation TMX on the MCF-7 cell line.

## Introduction

Breast cancer is the most common form of cancer among women with an incidence rate of more than 1.6 million per year (Siegel et al., 2018). Several organs such as bone, lung, liver, and lymph node are the most common and susceptible secondary sites for breast cancer metastasis (Chiang et al., 2008). In the process of metastasis when tumor cells enter the bloodstream directly or by the lymphatic system can alter the cellular composition, immune status, blood supply, extracellular matrix (ECM), and virtually every other aspect of the metastatic site to favor colonization leading to the formation of secondary niche. Among members of the MMP family, which are the zinc-dependent endopeptidases play a crucial role in the degradation of (ECM) associated with tissue repair, cancer cell invasion, metastasis, and angiogenesis. The members of the MMP family like MMP-2 (gelatinase -A) and MMP-9 (gelatinase-B) remain up-regulated in malignant tumors (Johnsen et al., 1998). TGFβ plays a crucial role in enhancing the migratory and invasive properties of cancer. A prerequisite property for the migration of epithelial cells demands the loss of cell-cell contact and development of fibroblasts characteristics which are known as the epithelial-mesenchymal transition (EMT). Accumulating documentation from several studies suggest that TGFβ is an established modulator of EMT. Another molecule that is also one of the key targets of repression during EMT is the cell-cell adhesion receptor E-cadherin. The deregulation of E-cadherin is commonly detected in many cancers, where its overexpression maintains the cell adhesion property and thereby reform the ECM network which helps suppress the invasion by tumor cells. It was found in many cancers that TGFβ induced EMT often coincides with the loss of E-cadherin expression (Miettinen et al., 1994a; Oft et al., 1996) whence, TGFβ potentially induces pro-angiogenic factors as result angiogenesis gets induced. Indeed, *in vitro* studies reveal that several key angiogenic mediators such as vascular endothelial growth factor (VEGF) and connective tissue growth factor (CTGF) are the direct targets of the TGFβ signaling pathway (Kang et al., 2003a; Sánchez-Elsner et al., 2001a). Therefore, the milieu of the metastatic phenomenon comprehends the most virulent property of malignancy that making the fiasco of the established therapy and leads to poor prognosis with low survivability. Therefore, in this context, it holds a great priority to constitute an affordable target-specific therapy with nominal toxicity. Naturally occurring compounds in this aspect can circumscribe these drawbacks of present therapy with a more valid distinguishable property such as economical than that of the conventional one. 

*Swertia chirata* is an Indian medicinal plant extensively used for diverse therapeutic purposes in ‘Ayurveda’. It possesses anti-helminthic, hypoglycemic, febrifuge, anti-malarial, anti-diarrheal, and antipyretic properties. Aqueous extract of *Swertia chirata* (whole plant) exhibited an inhibitory effect on the proliferation of human breast cancer cell T47D (Horwitz et al., 1982). The treatment with S. chirata extract found to inhibit the proliferation of prostate cancer PC-3 cells (Nawab et al., 2011). Our previous study indicated that 1,5,8-trihydroxy-3-methoxy xanthone (TMX) from *Swertia chirata* has anti-carcinogenic efficacy in mouse ascites tumor (Unpublished data), as the anti-metastatic potentiality of this compound has not been reported anywhere. In this paper, we evaluated whether TMX from S .chirata exerts anti-metastatic potential in the frame of *in vivo* and *in vitro* studies.

## Materials and Methods

*Chemicals*

Primary antibodies such as E-cadherin, α-tubulin, TGFβ, VEGF, MMP-9, and secondary antibodies like anti-mouse and anti-rabbit and luminal were purchased from Santa Cruz biotechnology USA. The EZcountTM MTT Cell Assay Kit for MTT was purchased from Himedia Laboratories. The TUNNEL assay kit was procured from Roche India. ELISA KITS were purchased from RandD BIOSYSTEMS SRL India and Real-time analysis was done by kit purchased from the Roche diagnostic. All the other chemicals used were of analytical grade and were purchased from common commercials.

*Plant material*

S. chirata (whole plant), collected from a local plant vendor Kolkata, and was verified of its genus by Dr. S.R. Das, Ex-Survey Officer, Central Research Institute (Ayurveda), Kolkata. The plant specimen has been conserved at the herbarium of the Central Research Institute (Ayurveda), Kolkata.

*Preparation of the extract*

The coarsely powdered aerial parts of *Swertia chirata* Buch. Ham (4kg) was extracted with normal hexane in soxhlet apparatus and the procedure was run for 72 hours. The extract was concentrated to afford a yellowish thick liquid (250 GM). This liquid was kept in the refrigerator for 24 hours. A pale yellow amorphous solid (2.4 GM) was separated from the child liquid. The solid was filtered and purified by repeated crystallization from ethanol. Finally, the yellow shining needle shaped crystals (TMX) were obtained (yield 0.52%) having MP . 270-271^o^C(Ghosal et al., 1978).

*Experimental animals*

Adult (5-6 weeks old) Swiss albino mice (25 ± 2 GM body weight) were obtained from our animal house (Chittaranjan National Cancer Institute, Kolkata, India). They were maintained at control temperature (23±2°C) and humidity (55±10 %) under alternating light and dark conditions (12 h/12 h). Animals were fed with standard food pellet diet and drinking water was provided regularly ad libitum. All the experimental procedures were carried out strictly following the guidelines of the Institutional Animal Ethics Committee (IAEC) following the CPCSEA Reg. No. -1774/GO/RBI/S/14/CPCSEA, India. 

*Non-toxic Dose Determination*

In the present study, animals were divided into six groups, containing six animals (n=6) in each group. Six sequential dosages of TMX (10, 20, 30, 40, 60 μg/kg body weight of mice) and a group that did not receive any treatment served as control and treated groups respectively. The experiments were performed in triplicate. The groups for this analysis were -

a) Normal which did not receive any treatment.

b) Treatment I received TMX orally at the dosage of 10 μg/kg body weight. 

c) Treatment II received TMX orally at the dosage 20 μg/kg body weight. 

d) Treatment III received TMX orally at the dosage 30 μg/kg body weight.

e) Treatment IV received TMX orally at the dosage 40 μg/kg body weight. 

f) Treatment V received TMX orally at the dosage 60 μg/kg body weight. 

To evaluate the toxicity, mice were treated consecutively for 14 days with the respective dosage for each group. On the 15th day, blood samples were collected from mice retro-orbital plexus. The levels of liver and kidney toxicity markers like SGOT, SGPT, Urea, Creatinine, were evaluated in an automated clinical chemistry analyzer (AU400, Olympus, Japan) according to the manufacturer’s protocol.

*Chronic toxicity study with non-toxic doses of TMX*

Chronic cytotoxicity was performed in normal mice (Andrade et al., 2014) using therapeutic, non-toxic dosage of TMX. Animals were divided into 2 groups containing six animals (n=6) in each group and the experiment was performed in triplicate. The groups for this analysis were 

a) Normal which did not receive any treatment.

b) TMX treated received TMX at the non-toxic dosage orally for 40days regularly.

Liver and kidney toxicity marker levels in the blood of normal mice were evaluated after 40 days of treatment by the automated clinical chemistry analyzer (AU400, Olympus, Japan) according to the manufacturer’s protocol. The drugs were given by oral administration of the therapeutic sub-lethal doses.

For the chronic toxicity experiments with the bone marrow of mice, bone marrow was flushed out using PBS and the pattern of cell cycle phase distribution with/without treatment of TMX were measured by Propidium Iodide staining followed by the analysis of fluorescence intensity in 1000 events by FACS (Calibur, Becton Dickinson, USA (Pal et al., 2012). The analysis was done using cell quest pro software.

*Bioavailability of TMX in normal mice*

Bioavailability of the drugs is the most important parameter for pharmacokinetics and pharmacodynamics study to execute proper therapeutic potential. In this study, six animals were orally fed with TMX at the non-toxic dosage and the experiment was performed in triplicate. Bioavailability of the TMX was measured by previously described method (Clifford et al., 2017) using LC-MS analysis in serum after taking the blood from the mice at different time point like 4 hours, 6 hours and 8 hours according to the manufacturer’s protocol.

*Development of EAC cell induced xenograft model*

Xenograft model is acclaimed for more clinical relevance in the tumor model. In our study, the xenograft model was developed by subcutaneous inoculation of 5x10^4^ EAC cells on the right flank of mice (Saraswati et al., 2013).

Day 0 and Day 40 were considered as the day of tumor cell transplantation and sacrifice respectively. The appearance of palpable tumors was noticed on the 8^th^-9^th^ day and mice were then grouped according to study design. 

The volume of solid tumors was measured using a slide caliper on a regular interval of every 7days after the appearance of palpable tumors. Upon visibility of palpable tumors, the mice were divided into experimental groups and each group contained 6 mice each. Each experiment was repeated thrice.

Control: Received only normal saline orally for 28 days. 

TMX treated: Received orally the non-toxic dosage of TMX for a period of 28 days.

All the *in vivo* experiments were performed in triplicate and there were n=6 mice in each group. All the results were represented as a standard error mean and was represented by the best representative picture.

*Cell culture*

Human breast adenocarcinoma cell line MCF-7, and non-cancerous cell lines like PBMC, HEK, and WI38 were purchased from NCCS, Pune, India, and cultured in DMEM media (pH 7.4) along with 10% FBS and antibiotics. MTT assay was performed to analyze the effect of TMX on MCF-7 and also the normal or non-cancerous cell lines *in vitro* according to the manufacturer’s protocol. All the in vitro experiments were performed in triplicate.

*Determinations of solid tumor size in Control and TMX treated tumor bearing mice*

The first palpable tumor was detected 7 to 10 days after tumor cell inoculation. A significant decrease in tumor volume was observed after treatment with sub-lethal dosage of TMX. Mice were sacrificed after 21 days from the day of treatment initiation with measurement of tumor volume and body weight in every alternate day after the first appearance of palpable tumors.

*Histopathological analysis of solid tumors and other organs*

Mice of different experimental groups were sacrificed following the experimental design. All the samples were fixed in 10% formaldehyde. Paraffin block was prepared following the standard protocol of our laboratory. About 4 μm thick paraffin sections were cut using Leica microtome RM40. After complete deparaffinization with Xylene followed by rehydration with a down gradation of alcohol, the sections were stained with hematoxylin and eosin (H and E) (Wu, 1940). The slides were examined under different magnification (10X, 20X, 40X) of the bright field microscope and consulted with the pathologist.

*Study of Phase-II detoxifying and antioxidative enzymes*

Phase II detoxification enzymes form the base of cellular defense mechanisms against carcinogens, toxic chemicals, and oxidative stress. Glutathione-S-transferase (GST) is a family of enzymes that helps catalyze the conjugation reaction of reduced glutathione. In the front of cellular protection against the oxidative stress, the classical antioxidant enzymes such as Glutathione peroxidase (GPx), Catalase (CAT), Superoxide dismutase (SOD) take major part along with those phase II detoxification enzymes.

In this study, the Phase II detoxification enzyme activity was measured in the solid tumor and liver of the xenograft model by previously described method (Habig et al., 1974; Johansson et al., 1988; Paglia et al., 1967).

*Effect of TMX on the generation of intracellular Reactive Oxygen Species (ROS)*

On the verge of maintaining the intracellular level of free radicals, a number of cellular defense mechanisms evolved to meet the need of maintaining a balance between generation and removal of this oxidative stress from cell. 

In this study, the relative level of ROS was measured by spectrofluorimetric analysis of bone marrow cells ,liver homogenate and solid tumor from the xenograft model following the method previously performed in our laboratory (Pal et al., 2012).

*Role of TMX on membrane peroxidation*

LPO was estimated in the microsomal fraction of hepatocytes and solid tumor using the thiobarbituric acid and protein was estimated by the conventional method (Ohkawa et al., 1979). The representation was done by illustrating thiobarbituric acid reactive substance (TBARS) formed per mg protein using the extinction coefficient of 1.56 × 10^5^ M^−1^ cm^−1^.

*Role of TMX on the expression of inflammatory and angiogenic markers*

The association between cancer burden and the expression of inflammatory markers are well known. In many cases, inflammatory responses become an integral factor of the tumor microenvironment, which helps to correlate with disease progression and prognostic aspect (Heidari-Soreshjani et al., 2017).

In this study, the expression of inflammatory markers was evaluated by the ELISA method. The serum was isolated from the blood sample as described previously. Single-cell suspension from the solid tumor was prepared by finely mincing samples in phosphate buffer saline followed by treatment with collagenase. The samples were then allowed to pass through a syringe and nylon mesh for repeated times.

The level of different inflammatory markers (IL-10, IL-1β, IL-4, IL-18) and angiogenic markers (VEGF, MMP-2, MMP-9) were analyzed in the single-cell suspension of solid tumor and in serum by ELISA method (Jiangsu Keygen Biotech Corp., Ltd) according to the standard protocol(Sporn et al., 1976).

*Expression analyses of different proteins by IHC, and Western Blotting*

In this study, IHC was performed following established protocol in our laboratory following the method described by Prince and Ginsberg, 1957, and performed on the solid tumors of the experimental groups. The protein expression pattern was scored according to (Perrone et al., 2006).

For immunoblot, cell and tissue lysates were prepared with RIPA buffer. The total protein lysate was separated by SDS-PAGE. The immunoreactive protein bands on the membranes were visualized using chemiluminescence reagents (Millipore) (Burnette et al, 1981).

*Cell death detection (apoptosis) in situ*

Apoptotic cells in the solid tumor were visualized using the terminal deoxynucleotidyltransferase (TdT) mediated dUTP-biotin nick end labeling (TUNEL) method with the help of in situ cell death detection kit, POD (Roche Molecular Biochemicals). The deparaffinized tissue sections were permeabilized using Triton-X-100 (Sigma, USA). The sections were then incubated with a TUNEL reaction mixture containing TdT and fluorescein dUTP, at 37 0C for 30 min, rinsed in PBS for 10 min, it was incubated in POD converter at 37 0C for 30 minutes at visualized in brightfield microscope (Leica) after staining with DAB following standard procedure (Saha et al, 2004).

*Cytogenetic analysis*

Chromosome preparation from bone marrow cells of tumor bearing mice in both control and treated groups was performed by following the mitotic division inhibition technique in practice in this laboratory (Mallick et al., 2018). The technique is described in brief as below:

i) Both the control and treated specimens received an injection of 0.04% colchicine solution intraperitoneally at a rate of 1 ml/100 g body weight for 2 h. ii) Cells from bone marrow were collected in a hypotonic solution (0.075 M KCl) and aspirated gently. Then the cell suspension was incubated for 30 min at 37 °C and centrifuged for 15 min at 1500 RPM to collect the cell sediment. The sediment was fixed in acetone-alcohol fixative (3:1, methanol: glacial acetic acid, v/v) and centrifuged for 8 min. The sediment was fixed again and kept for chromosomal slide preparation. The flame dried prepared slides were stained with 5% Giemsa diluted in phosphate (pH 6.8) buffer. Only well-spread metaphases were analyzed under the bright field microscope (10*100x magnification).

*In vivo validation of metastasis*

Bone marrow from solid tumor bearing mice both TMX treated and untreated were injected into the right flank of fresh and normal mice to observe the tumor development.

*Cell migration assay*

MCF-7 cells were seeded in 35mm plates and allowed to grow for 80% confluence in a single layer. A scratch was wounded on the cell bed with a sterile 200µl pipette tip along the diameter of the plate. The cells were then treated with TMX and incubated for 24h and 48h. After incubation, the cells were washed with 1X PBS to remove cell debris and the scratch was visualized under a microscope and photographed at 40X magnification under bright-field microscopy. The experiment was performed in triplicate.

*Colony formation assay*

MCF-7 cells were treated with TMX for 48 hours and plated in a six-well plate using a 3 ml complete growth medium at a density of 1,000 cells/well. After ten days of incubation, the cells were stained with a crystal-violet solution [0.05% (w/v) crystal violet in 20% (v/v) methanol] for 10 min, and colonies were counted under the bright-field microscope. The experiment was performed in triplicate.

*Quantitation of mRNA of different genes*

RNA was isolated from the solid tumor using Roche high pure RNA isolation kit according to the manufacturer’s protocol. cDNA synthesis and real-time analysis were performed by the cDNA synthesis kit and FastStart Essential DNA Green Master from Roche life science respectively following the manufacturer’s protocol.

*Statistical analysis*

All the experiments encompassing* in vivo* and *in vitro* were performed in triplicate. In the case of *in vivo*, each experiment were performed thrice and there were n=6 mice in each group. All the data were represented as mean ±SD. All the calculations were done using MS-Excel software. The Results are expressed as mean ±SD or as a percentage or as a relative expression level. The data from all the quantitative assays were analyzed and quantified using unpaired Student’s t-test and a P-value of <0.005 was considered significant.

## Results

*Determination of the non-toxic dose of TMX*

**Figure 1:**
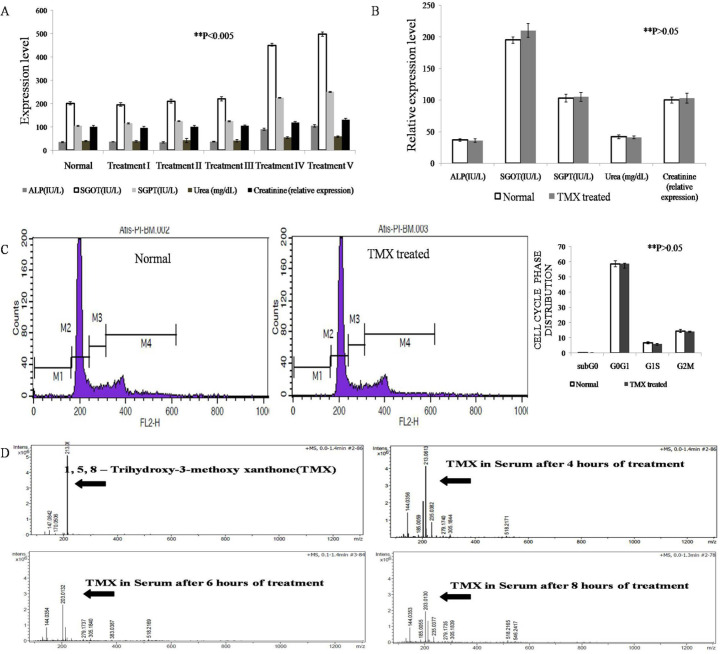
A. Sub acute toxicity screening of TMX in normal swiss albino mice.Among the range of dosage screened, 20µg/kg was found to be non-toxic when tested for sub acute toxicity in normal swiss albino mice. B. TMX dosage 20µg/kg was found to be non-toxic when tested for chronic toxicity as no significant difference in liver and kidney toxicity parameters were found between Normal and TMX treated group P>0.05. C. Cell Cycle Analysis of bone marrow cells following chronic exposure to TMX at therapeutic dosage 20µg/kg concentration for 40 days. No significant difference was observed between cell cycle phase distribution of bone marrow cells of Normal and TMX treated group of mice P>0.05. D. Bioavailability study of serum by mass spectrometry analysis. With increasing time the 213KD peak indicating TMX was present up to 8 hours

Non-toxic dose determination was performed by treating the normal mice with different doses of TMX (5µg/kg, 10µg/kg, 20µg/kg, 40µg/kg body weight). The acute toxicity study showed dosage 40µg/kg body weight showed a significant increase in biochemical and hematological toxicity parameters in comparison to untreated mice, whereas the dose of 20µg/kg did not show any significant difference in comparison to normal (Figure 1A). Upon performing a chronic toxicity study with dosage of 20 µg/kg no toxicity was detected with respect to normal mice (Figure 1B). The findings of the chronic study were validated by cell cycle analysis of bone marrow of mice from the chronic study group where no variation in the cell cycle phase distribution of bone marrow cells of Normal and TMX treated mice was seen (Figure 1C). Therefore, the dosage 20µg/kg was selected as a therapeutic dose for further investigations.

*Bioavailability of TMX in mice*

Bioavailability study was performed in mass spectrometry using serum collected from the blood samples after 4th, 6th, and 8th hour of treatment with TMX of 20µg/kg. The purified compound was found as a single peak at the 213gm/mole. We also found the peak at the position of the same molecular weight in the serum of TMX treated mouse (Figure 1D). Thus, it was concluded that TMX is biologically available even after 8 hours of treatment.

*Restriction of the development of solid tumor after TMX treatment*

**Figure 2:**
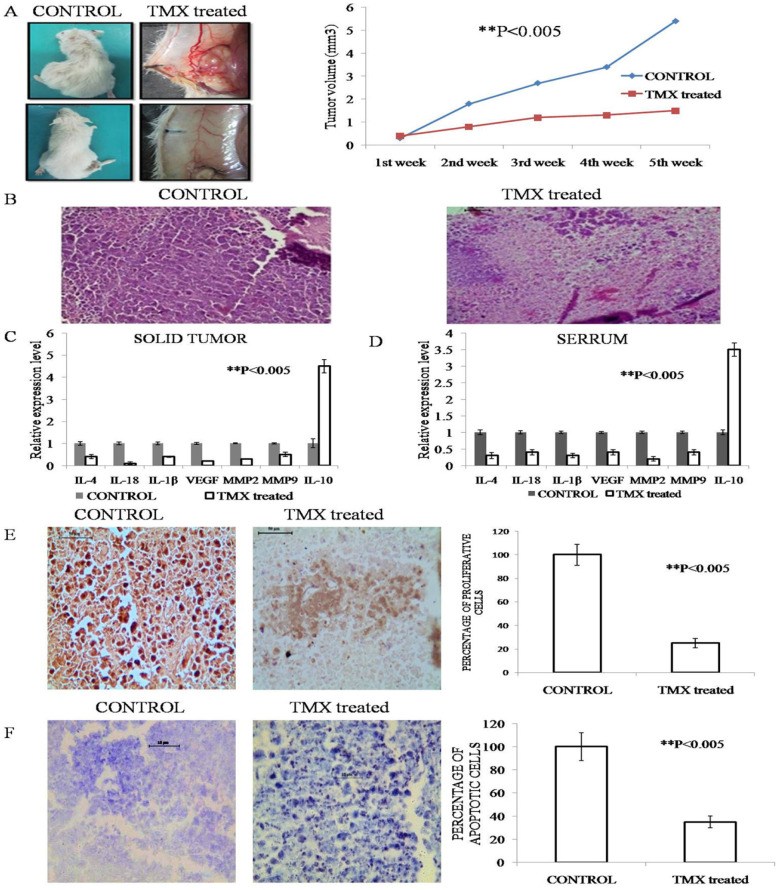
A. Restriction of tumor growth by TMX treatment in the experimental groups Control and TMX treated. Size and pattern of vasculature were reduced in treated group in comparison with control. Data represented as mean ±SD with significance **P<0.005; B. Representative histological photographs of solid tumor where tumorigenic cell number reduced in the TMX treated sample. Photographs taken under 20X magnification of the bright field microscope; C. Graphical representation of relative expression of pro and anti-inflammatory and angiogenic markers. Solid tumor samples of the experimental groups CONTROL and TMX treated mice where upregulation was observed only in anti-inflammatory factor IL-10 whereas all the other parameters reduced upon TMX treatment. Data represented as mean ±SD with significance **P<0.005. D. Graphical representation of relative expression of pro and anti-inflammatory and angiogenic markers. Serrum samples of the experimental groups CONTROL and TMX treated mice where upregulation was observed only in anti-inflammatory factor IL-10 whereas all the other parameters reduced upon TMX treatment. Data represented as mean ±SD with significance **P<0.005; E. Representation of PCNA expression by IHC analysis of the experimental groups CONTROL and TMX treated. Representative photographs showed reduced PCNA expression in treated group in comparison with control. Photographs taken under 20X magnification of the bright field microscope. Graphical representation showed percentage of proliferative cells. Data represented as mean ±SD with significance **P<0.005; F. Representation of in situ TUNEL assay (apoptotic population) in the experimental groups Control and TMX treated. Representative microscopic photographs of TUNEL positive cells were indicated by arrow. Photographs taken under 20X magnification of the bright field microscope. Increased cell death was found in TMX treated group with respect to CONTROL. Graphical representation showed the percentage of apoptotic population in the experimental groups. Data represented as mean ±SD with significance **P<0.005

EAC cells were injected in the right thigh of mice at a dilution of 1×10^5^, collecting from the 8-10 days old ascites bearing mice. After the appearance of palpable tumors around 8/9 days, the size of the tumors was measured in every alternate day with a slide caliper as per established protocol (Jaganathan et al., 2010). The graph represented the significant restriction (**P<0.005) of the volume of tumor around 1.5mm3 when compared with the untreated Control with an average volume of 5 mm^3^ (Figure 2A).

The growth of the tumor was demonstrated by sacrificing the mice on the 40^th^ day and performing histological analysis. The infiltrating tumor cells with a high n/p ratio along with infiltrating lymphocytes (TIL) were prominent in histological analysis, indicative of an aggressive tumor (Figure 2B).

*Effect of TMX on blood and biochemical parameters of xenograft tumor bearing mice*

The observation of the Hematological and biochemical parameters delineated overall protection of mice’s health which was fetched up by the malignant condition of solid tumor. The increased level of WBC and PLT count was brought back to normal when TMX treatment was administered concerning control at the non-toxic dose as well as RBC and HGB level was regulated in such a way it became closer to normal. A Similar trend of TMX mediated modulation was also observed in the biochemical parameters where all the elevated levels reduced significantly (**P<0.005) in comparison with control (Table1).

*Cytoprotective role of TMX on xenograft tumor bearing mice*

The result of the increase in Phase –II detoxification enzymes simultaneously with the minimizing intracellular level of oxidative stress again revealed the fact that TMX systematically boosted the host detoxification system when carcinoma cell was implanted followed by its progression into the tumor. A complete reverse pattern of the alteration of the markers (**P<0.005) was observed in the solid model that warranted TMX mediated cytotoxicity to cancer cells in malignant condition (Table1).

*Analysis of Inflammatory and angiogenic markers in both serum and tumor samples*

As to the mechanism underlying such protective effects, TMX may function by suppressing inflammation. The expression pattern of inflammatory factors in the tumor and blood was examined by ELISA. As shown in (Figure 2C-2D), pro-inflammatory factors IL-1β, IL-4, and IL-18 were significantly up-regulated while anti-inflammatory factor IL-10 is significantly down-regulated in the TMX treated group when compared with the untreated Control group. Treatment with TMX not only inhibited the expressions of pro-inflammatory factors but also promoted those of the anti-inflammatory factors suggested its anti-inflammatory properties. The inflammation plays a triggering role in the process of angiogenesis, therefore, some markers related to angiogenesis such as VEGF, MMP-2, MMP-9 were analyzed and found to be significantly downregulated due to the application of TMX (Figure 2C-2D) (**P<0.005).

*The effect of TMX treatment on cell proliferation and apoptosis*

A significant reduction in the number of proliferative cells after TMX treatment was evident from BrdU incorporation assay in the slides of control and TMX treated solid tumors. A significant increase in the number of tunnel positive cells was observed after TMX treatment in the solid tumor slides of the same experimental groups (**P<0.005) (Figure 2E -2F).

*Role of TMX on the restriction of EAC cells’ metastasis to the lung and liver of solid tumor bearing mice by Histopathological analysis*

**Figure 3:**
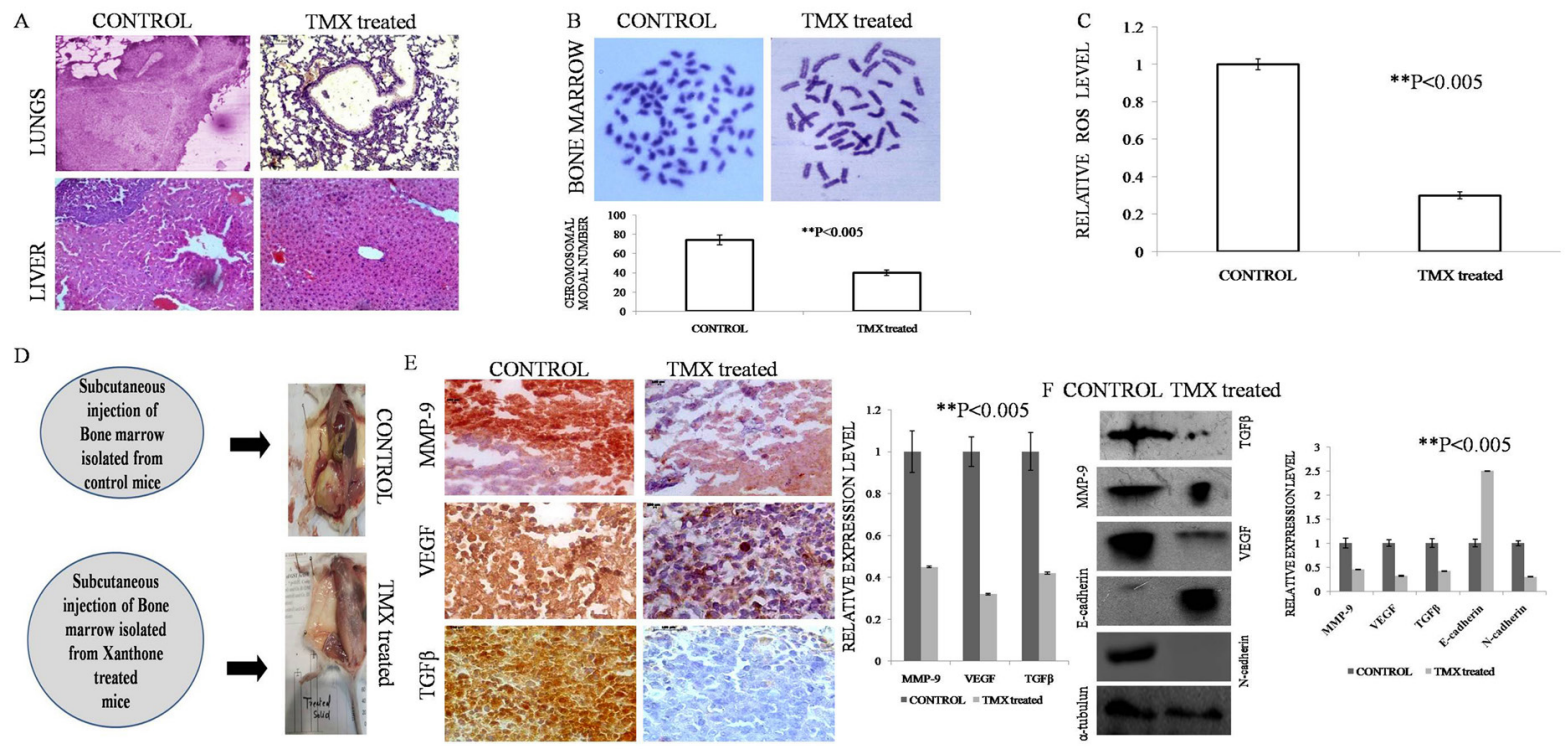
A. Histological investigation, revealed infiltrating tumor cells causing cancerous chages in lung and liver of CONTROL group of mice, whereas no such infiltrating cells was observed in TMX treated group. B. Increase in the chromosomal modal number in the bone marrow was observed in the CONTROL group of mice, whereas no change in the modal number of the TMX treated group was observed. (P<0.005). C. Intracellular ROS of bone marrow in solid tumor bearing mice was found to be significantly higher as compared to the TMX treated (P<0.005). D. Upon injection of the bone marrow of the solid tumor bearing mice on the right flank region of the fresh mice, we see the formation of an aggressive tumor whereas no such tumor formation was seen upon injection. E. Representation of TGFβ, MMP-9, VEGF expression by IHC analysis of the experimental groups Control and TMX treated. Representative photographs showed reduced TGFβ, MMP-9, VEGF expression in treated group in comparison with control. Photographs taken under 20X magnification of the bright field microscope. Graphical representation showed percentage of proliferative cells. Data represented as mean ±SD with significance **P<0.005. F. Expression of metastasis related genes by western blot of the experimental groups CONTROL and TMX treated in EAC solid tumor bearing mice. Densitometric analysis through graphical representation showed relative expression of individual markers. Peak density was normalized by the loading control GAPDH. Data represented as mean ±SD with significance **P<0.005

During the progression of aggressive tumorigenesis, the cancer cells generally migrate in the probable distant metastatic sites such as lung, liver, and bone marrow. In this study also the histological evaluation revealed severely magnified metastatic potential in the Control group of solid tumor sample while it was found highly restricted in the TMX treated sample. The detailed observations are listed below (Figure 3A).

*Control liver*

The morphology of EAC control liver showed severe hyperplasia with an increased number of cells and loss of radial distribution pattern of cells around the central vein where most cells showed n: p>1. Therefore, it indicated infiltration of the cells from the solid tumor due to metastasis caused hyperplasia in liver.

Treated liver treated liver: The histological analysis of TMX treated liver showed almost normal morphology of cells and the infiltration of the cells was negligible. The radial distribution of cells surrounding the central vein was also present.

*Control lung*

The histological analysis of control lung revealed a higher number of cell proliferation and formation of a solid mass, hence it indicated that the Control lung possessed carcinoma due to metastatic cells from the solid tumor.

*Treated Lung*

The histological analysis of the treated lung showed the morphology closely resembled the normal architecture, however, the columnar epithelial lining was slightly lost. Hence, it revealed TMX intervention significantly restricted the metastatic potential of solid tumor cells. 

*The effect of TMX treatment on Bone marrow of solid tumor bearing mice*

The modal number of the bone marrow in solid tumor bearing mice was found to be 74 which was indicative of metastatic colonization of EAC induced solid tumor. On the TMX treatment, the modal number of chromosomes was found to be 40 which is the normal chromosome number of mice (Figure 3B). In addition, the chromosome of Solid tumor bearing mice showed increased intracellular ROS level while TMX treatment caused a significant lowering of ROS level (Figure 3C).

*In vivo validation of restriction of metastasis upon TMX treatment*

On injecting the bone marrow cells both from the Control and TMX treated mice kept flushed and collected in 1X PBS previously into the right flank of fresh mice, the formation of the solid tumor was thoroughly noticed. The tumor formation in the mice group with the bone marrow cells collected from the Control group of mice resulted in a very large volume of the tumor. Whereas, the result surprisingly revealed with the absence of tumor formation in the mice which were injected with the bone marrow cells collected from TMX treated group of mice. 

Hence, from this analysis, it was inferred that in the source mice (bone marrow cells were collected) the TMX treated group of tumor cells lost their migratory property so that the lack of EAC tumor cells in the bone marrow failed to form any tumor in the fresh mice as opposed to the scenario observed with the EAC containing bone marrow cells collected from the Control group of mice (Figure 3D).

*Inhibitory effect of TMX on the expression of metastatic markers*

In the next phase of validation of the inhibitory effect of TMX on the metastatic property, the expression of several metastatic markers was evaluated through IHC and Western blot.

The expression of MMP-9, VEGF, TGFβ was found significantly reduced (**P<0.005) after TMX treatment observed in the solid tumor sample of IHC analysis than that of the Control samples.

A similar pattern of expression was found when validation of expression was performed by Western blot analysis. Expression of all the aforementioned markers tethered significantly with a significant increase of E-cadherin and decrease of N-cadherin expression in TMX treated lysate of the solid tumor when compared with untreated control with the level of significance **P<0.005 (Figure 3E-3F).

Hence, from the series of analysis, it was proved that TMX potentially impeded the phenomenon of metastasis *in vivo* against EAC induced solid tumor.

*In vitro evaluation of anti-metastatic potential of TMX against breast carcinoma cell line MCF-7*

Each in vitro experiment was performed thrice and the results were represented ±SD.

*Effect of TMX on cytotoxicity on cancer cells*

As seen in the MTT assay TMX treatment effectively inhibited breast adenocarcinoma cells MCF-7 at the lowest concentration of 3µM whereas it did not show any effect on normal cells such as PBMC, HEK, and WI38 with the highest dosage of 100µM (Data not shown). 

Therefore, this study showed the specific toxicity towards cancer cell line at 3 µM that was used as the IC50 dose for further analysis. Hence, the inefficiency of cytotoxicity of TMX towards normal cell line reciprocated as the absence of side effects.

*In vitro validation of the effect TMX on the restriction of the migratory property of MCF-7 cancer cells*

**Figure 4:**
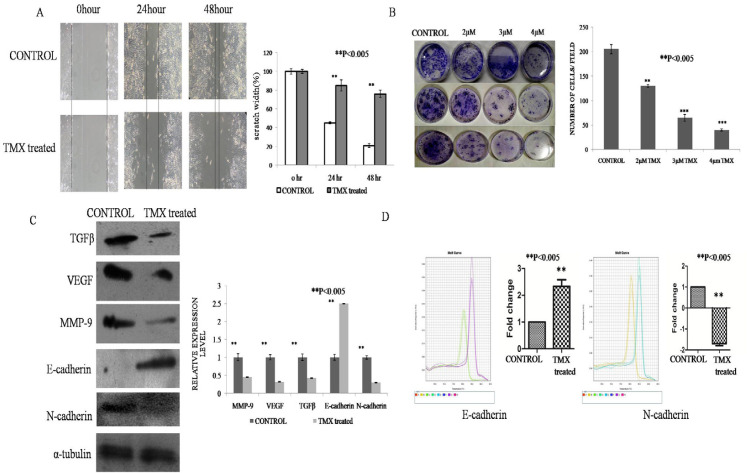
A. Cell Migration Activity Determined by Wound Healing Assay in MCF-7 Cell following exposure to TMX at IC50 (3µM) Concentration for 24 and 48 Hours. The data obtained for TMX are compared with control. Each bar represents ± SD of the triplicates. The level of significance was set at **P<0.005. B. Colony forming ability of MCF7 at concentrations 2µM, 3µM and 4µM of TMX with respect to untreated control and a sequential decrease in the number of colonies was observed. Assays were performed thrice quantification was performed by manual counting. Each bar represents ± SD of the triplicates. The level of significance was set at **P< 0.005. C. Expression of metastasis related genes by western blot of the experimental groups Control and TMX treated. Densitometric analysis through graphical representation showed relative expression of individual markers. Peak density was normalized by the loading control GAPDH. Data represented as mean ±SD with significance **P<0.005. D. Relative mRNA expression of E-cadherin and N-cadherin by quantitative RT-PCR. GAPDH was used as an endogenous control to normalize the expressions. Data represented as mean ±SD with significance **P<0.005

In the study of the scratch assay, The Control group showed significant migratory property after 24 hours and scratch was almost completely healed at the end of 48 hours. TMX treatment showed significant restriction on the migratory property of the MCF 7 cell line at the end of 24 hours and highest at the end of 48 hours. Increase in restriction was attributed by keeping the width of the scratch as same as the beginning by inhibiting the migration of cells. Thus, our results suggested that TMX could restrict the migration process (Figure 4A).

*Effect of TMX on the restriction of colony-forming ability of cancer cells*

The role of TMX on the restriction of the mobility of MCF-7 cells was validated by their colony-forming ability or clonogenic assay. The result showed the ability of colony formation of the MCF-7 cells significantly decreased in a dose-dependent manner after treatment with the IC_50_ dose of TMX in comparison to untreated Control. In control plates, an average of 200 colonies was observed per plate. Upon application of 2µM TMX, the number of colonies reduced to around 130 and the treatment with IC_50_ value 3µM showed an even lower number of colonies around 65. TMX 4 µM treatment caused further decreased in the colony number to around 40. This assay was performed thrice and the graph was represented as mean ± SD (Figure 4B). 

*Molecular mechanism of restriction of metastasis by TMX treatment in vitro*

To explore the molecular mechanisms of restriction of metastasis of cancer cells after TMX treatment, the protein expression profile of different regulatory genes involved in the migration of MCF-7 cells was performed by Western blot analysis. It was found from our result that overexpression of E-cadherin and downregulation of TGFβ, VEGF, MMP-9 and N-cadherin level in the TMX treated sample in comparison to Control which was significant P<0.005 (Figure 4C). Each western blotting was performed thrice. Data were represented as mean ±SD with significance.

The *in vitro* validation of the restriction metastasis was performed by real-time PCR. A significant decrease in the N-cadherin and a significant increase in the E-cadherin transcript level were observed after TMX treatment (**P<0.005) (Figure 4D). Real-time PCR experiment was performed thrice. Data were represented as mean ±SD with significance.

Therefore, it was proved from our study that by modulating the metastatic markers at the transcriptional level, TMX restricted the propagation of EMT and simultaneously maintained the cell-cell junction hence the adherence property, so that the ECM matrix meshwork retained its structure and restricted the cell migration.

**Table 1:** Detoxifying Enzymes, Endogenous LPO and ROS in Liver and Solid Tumor

Groups	GSH	GST	Gpx	SOD	CAT	LPO	ROS (Relative)
	(nmol/mg)	(nmol/mg)	(nmol/mg)	(unit/mg)	(Unit/mg)	(moles/mg)	
Sample: Liver							
Control	0.8 (±.02)	1.75 (±0.46)	2.5 (±.075)	6.29 (±1)	1.4 (±0.1)	9.5 (±1.2)	1
TMX treated	2.72 (±1)**	12 (±3) **	2.9 (±0.9)**	40 (±1.8)**	3.7 (±0.1)**	1.8 (±0.2) **	0.35 **
Sample: Solid Tumor							
Control	20 (±0.8)	1.75 (±0.4)	2.5 (±.08)	47 (±0.8)	17 (±0.6)	5 (±1.2)	1
TMX treated	17 (±1) **	0.56 (±.05) **	0.9 (±2) **	35 (±0.7) **	8 (±0.1) **	15 (±.09) **	4 **

** Significant difference in the TMX treated group in comparison with Control group considering p<0.005; Table 1 Comparative study of modulation of Detoxifying enzymes, endogenous LPO and ROS in liver and solid tumor of control and TMX treated group of mice. Values represent ±SD of six samples in each group.

**Table 2:** Primer Sequences Used for Real Time PCR *in vitro* Study

Name of gene product	Primer sequence
E-cadherin (Human)	Forward
	GCTCTGAGGAGTGGTGCATT
	Reverse
	GCAATTTCTCGGCCCCTTTC
N-cadherin (Human)	Forward
	AGGGGAGAGGTGCTCTACTG
	Reverse
	GGGGTAATCCACACCACCTG
GAPDH (Human)	Forward
	GCAACTAGGATGGTGTGGCT
	Reverse
	TCCCATTCCCCAGCTCTCATA

Primer for real time PCR designed from NCBI primer blast

## Discussion

In recent years, there has been a high increase in the identification of active compounds from natural products due to their lower toxicity compared to conventional therapy and ease of affordability though the rate of these natural products being translated into a marketable one is not high enough. The set back that makes it difficult to being a commercial therapeutics is its poor bioavailability. The three important categories that assist a natural compound to become a potential bioactive one availed by several drug delivery systems are *in vivo* bioavailability, pharmacokinetics, and first-pass metabolism reported in the case of curcumin, quercetin, and wogonin (Grill et al., 2014). 

Our study with TMX isolated from *Swertia chirata* showed higher bioavailability that made it secure promising anticancer ability with no notable toxicity on the both *in vivo* and *in vitro* system when tested for chronic toxicity and cell viability assay respectively. Furthermore, the anticancer activity was proved by remarkably restricting the solid tumor volume at the non-toxic dosage of TMX indicated clenching a promising therapeutic potential. The higher expression of interleukin IL-1β and IL-4 delineated a high-risk association with bone metastasis and tumor progression (Tulotta et al., 2018). Our study also corroborated this fact of increased IL-1β and IL-4expression that was justified by increased bone marrow chromosomal modal number, indicated metastatic property of EAC induced solid tumor to bone marrow and increase in the size of solid tumor although an extreme reverse scenario was observed when the system was intervened with TMX that favored utmost restriction of metastasis and tumor volume. In contrast to IL-1β and IL-4, IL-10 overexpression elicits tumor rejection by stimulating cytotoxicity of CD8 (+) T cells by inducing the expression of IFN-γ in CD8 (+) T cells(Oft et al., 2014). The treatment with TMX significantly increased IL-10 production and thereby stimulated antitumor immune response of the host body. The metastatic cancers target different secondary organs that are often difficult to treat and demand partial or complete resection of affected organs. Therefore, a drug targeting metastatic cancer is the need of the hour. The organs such as bone, lung, and liver are the most common target organs for the metastasis of breast cancer cells (Kennecke et al., 2010), Similarly here in our study, the untreated Control mice showed metastasis to bone, lung, and liver, which was effectively restricted upon TMX treatment. *In vitro* validation of the anti-metastatic property of TMX was achieved by inhibiting the migratory and the colony-forming ability of MCF-7 cells upon TMX treatment. In the process of normal development, Epithelial to mesenchymal transition (EMT) is clearly evidenced albeit the aggressive cancers exploit this phenomenon fully as a trigger to propagate the malignant cells from primary sites to several secondary organs termed as metastasis (Tomaskovic-Crook et al., 2009). Cancer cells that undergo metastasis survive in the metastatic phase and are more robust than the other cancer cells and seem to be the main reason behind the recurrence and drug-resistant phenotype of most cancer (Radisky et al., 2005). It is also known from previous studies that, undifferentiated cancer cells own metastatic properties and impart robust characteristics to cancer cells (Kirsten et al., 1987). There are several key players orchestrating the phenomenon of metastasis. Overexpression of marker like MMP-9 is linked to the rapid progression of tumorigenesis and poor overall survival of cancer patients due to metastasis. Interestingly, the TGFβ pathway has been shown to play a role in various metastatic processes by modulating the ability of tumor cells to spread throughout the body (Bhowmick et al., 2004; Pollard et al., 2004). TGFβ can enhance the migratory, angiogenic, and invasive properties of cancer by inducing EMT. The other molecular marker E-cadherin is one of the key targets of repression of the process of EMT and is commonly deregulated in many cancers, over expression of which can suppress invasion by tumor cells. Accumulating evidence suggest that EMT induced by TGFβ, leads to the loss of E-cadherin expression (Miettinen et al., 1994; Wels et al., 2008). Previous studies illustrated that N-cadherin being up-regulated in more invasive and undifferentiated breast cancer cell lines resulted in severe repression of E-cadherin expression (Hazan et al., 1997). Reports showed that undifferentiated breast cancer clones possessing higher N-cadherin expression elicit the ability of confirmed liver metastasis (Kern et al., 1994). Various *in vitro* studies established that angiogenic factors such as VEGF and connective tissue growth factor (CTGF) are direct targets of the TGFβ signaling pathway as it enslaves the hypoxic condition present at the core of tumor leading to induction of a robust level of VEGF mRNA (Kang et al., 2003b; Sánchez-Elsner et al., 2001β;Ziello et al., 2007). In our study, both *in vivo* and *in vitro* model showed considerable augmentation of metastasis in the Control group with significantly upregulated expression of TGFβ, VEGF, MMP-9, N-cadherin, and a severe downregulation of E-cadherin. TMX treatment reversed the expression of these key markers of metastasis which fosters the basis of the chemotherapeutic potential of TMX.

Therefore, in the conclusion, the *in vivo* and *in vitro* studies warranted the TMX counterweighed the metastatic potential of breast adenocarcinoma cell lines thereby effectively overcame the drawback of present therapies. Hence, this study invigorated the role of TMX as a potent future therapeutics against metastatic breast cancer in a substantial cost-effective manner.

Therefore, we can say that TMX could exert its therapeutic property by restricting metastasis.
